# Development and effectiveness test of an internet-based real-time monitoring device for malaria parasite vector mosquitoes

**DOI:** 10.1371/journal.pntd.0014778

**Published:** 2026-10-09

**Authors:** Chenyu Han, Yiquan Cai, Huijuan Yang, Yiji Li, Xuejian Zhang, Xiaoming Wang, Yixuan Duan, Hanren Liu, Lei Zuo, Guiyun Yan, Xiao-Guang Chen

**Affiliations:** 1 Department of Pathogen Biology, Institute of Tropical Medicine, School of Public Health, Southern Medical University, Guangzhou, Guangdong, China; 2 Key Laboratory of Tropical Translational Medicine of Ministry of Education, School of Basic Medical Sciences, Hainan Academy of Medical Sciences, Hainan Medical University, Haikou, Hainan, China; 3 Chongqing Wanzhou District Center for Disease Control and Prevention, Chongqing, China; 4 Department of Population Health and Disease Prevention, Joe C Wen School of Population and Public Health, University of California at Irvine, Irvine, California, United States of America; Egerton University, KENYA

## Abstract

**Background:**

Malaria remains a major global health threat, and effective vector surveillance is essential for timely vector control. However, surveillance systems are challenged by behavioral shifts in *Anopheles* mosquitoes, labor-intensive procedures, and delays in real-time data transmission. Existing electronic monitoring tools often suffer from low sensitivity and specificity, creating an urgent need for automated, high-precision alternatives.

**Methods:**

We developed the “Black Box,” a 3D-printed, internet-enabled device integrating multispectral light, thermal simulation, chemical attractants, and photocatalytic materials with automated counting and wireless data transmission. Laboratory trials demonstrated trapping rates of 94.00%–95.00% for *Anopheles sinensis*, *An. stephensi*, and *An. anthropophagus*, with data consistency exceeding 97.00%. Semi-field tests yielded trapping rates of 73.60%–90.80% and consistency above 92.00%. In field deployments, the device captured 5,109 mosquitoes with a 94.17% consistency rate and captured more *Anopheles* than the light trap during the field observation period. Real-time data revealed distinct bimodal activity peaks at dawn and dusk. Among the 112 field-captured female *An. sinensis*, 21 (18.75%; exact 95% CI, 12.60%-26.97%) were blood-fed, indicating that blood-fed mosquitoes may occur in outdoor collections.

**Conclusions:**

The Black Box provides a highly efficient, automated solution for real-time malaria vector surveillance. By delivering accurate, timely ecological data, it supports dynamic risk assessment and enables targeted interventions, addressing key limitations of current control programs.

## Author summary

Monitoring malaria-vector mosquitoes is essential for directing control measures, but commonly used methods require substantial field labor, may expose collectors to mosquito bites, and often delay the availability of surveillance data. We developed the Black Box, an internet-connected mosquito trap that combines light, heat, chemical attractants, photocatalytic material, automated counting, and wireless data transmission. We evaluated the device under laboratory, semi-field, and field conditions. It captured three major malaria-vector species efficiently in controlled experiments and maintained close agreement between physical mosquito counts and records transmitted to the cloud platform. During field deployment, the device continuously recorded mosquito activity and revealed higher activity at night and peaks around dawn and dusk. These data show that an automated system can provide timely information on mosquito abundance and activity while reducing dependence on repeated manual counting. The current device cannot identify individual *Anopheles* species automatically, so captured mosquitoes still require morphological examination. Further development and testing across seasons and regions will be needed before species-level automated identification and wider operational use can be established.

## Introduction

Malaria is an infectious disease caused by *Plasmodium* parasites and transmitted by *Anopheles* mosquitoes, including *An. gambiae*, *An. sinensis*, *An. stephensi*, and *An. anthropophagus* [1–3]. It endangers the lives and health of hundreds of millions of people worldwide each year and imposes a great economic burden on social development [4]. Vector management, particularly through the use of long-lasting insecticidal nets (LLINs) and indoor residual spraying (IRS), is a cornerstone of malaria control aimed at suppressing endophilic (indoor-active) *Anopheles* mosquitoes [5–8]. However, the long-term and extensive use of insecticides has led to widespread insecticide resistance in vector *Anopheles* mosquitoes, accompanied by ecological behavioral changes, for example, shifting from endophilic to exophilic (outdoor-active) behavior [9]. This renders traditional vector control methods ineffective, making the control of exophilic *Anopheles* mosquitoes a major challenge in malaria prevention and control.

Currently, common monitoring methods for malaria vectors include human landing catches (HLC), light traps, and tent traps [10]. However, each of these conventional surveillance methods has distinct practical limitations. HLC requires on-site operation by trained personnel, which incurs high labor costs and exposes collectors to pathogen-infected mosquito bites, making large-scale, high-frequency long-term monitoring unfeasible [11]. Light traps exhibit poor trapping selectivity for *Anopheles* mosquitoes and frequently capture numerous non-target insects, introducing substantial noise into datasets and imposing heavy workloads for subsequent morphological species identification [12]. Tent traps are highly susceptible to ambient temperature, humidity, wind speed, and operator proficiency, resulting in poor data comparability and low standardization across sampling trials [13]. More critically, all traditional approaches rely on manual mosquito counting and paper-based recording, leading to substantial delays in data aggregation and transmission that fail to meet the real-time data requirements for early warning of mosquito-borne disease outbreaks [14,15]. In recent years, internet-based real-time monitoring technologies have provided new solutions for the prevention and control of mosquito-borne diseases [16–18]. By deploying intelligent monitoring devices, data such as mosquito density [19], species [20], and activity patterns [21,22] can be collected in real-time. Combined with geographic information systems (GIS) and data analysis technologies, dynamic assessment and early warning of vector-borne diseases can be achieved, providing a scientific basis for precise control. For instance, Biogents (Biogents GmbH) has recently developed the Biogents BG-Counter 2M Remote Mosquito Surveillance Device, which uses an infrared detection system to automatically identify mosquitoes and transmit captured mosquito data and environmental variables to the database and terminals [23,24].

Their findings indicate that the monitoring efficacy of the BG-Counter 2 is inconsistent: while it achieved a median accuracy of 89.0% in urban settings, the median accuracy dropped significantly to 20.6% in rural test sites. Furthermore, *Anopheles* mosquitoes accounted for only a minor fraction of the total captures. Consequently, the sensitivity and specificity of this device still require substantial improvement.

In summary, the current surveillance system for malaria vectors faces multiple bottlenecks. Conventional manual surveillance methods demand intensive manpower and produce data with substantial time lags. Commercially available intelligent traps suffer from insufficient recognition sensitivity and poor trapping compatibility with *Anopheles* mosquitoes. Although the automated monitoring device previously developed by our group enables stable real-time surveillance of *Aedes* and *Culex* mosquitoes, it lacks effective detection performance for *Anopheles* species. Moreover, its single trapping mechanism cannot accommodate the diverse resting preferences and activity rhythms of *Anopheles* mosquitoes across indoor and outdoor microhabitats. To address these technical limitations, we developed an internet-based real-time and efficient monitoring device for malaria vectors, by integrating multiple mosquito-attracting factors (e.g., specific wavelength light sources, temperature simulation, chemical lures, and photocatalytic materials) with automatic mosquito identification and wireless transmission technologies. The performance of this device was evaluated under laboratory, semi-field, and field conditions.

## Methods

### Mosquito strains and rearing conditions

*Aedes albopictus* (*Ae. albopictus*) and *Culex quinquefasciatus* (*Cx. quinquefasciatus*): Collected in Foshan, Guangdong Province by the Guangdong Provincial Center for Disease Control and Prevention in 1981, these strains have been standardized and maintained in the Laboratory of Pathogen Biology, Southern Medical University to date. *An. sinensis* and *An. anthropophagus* were obtained from the Wuxi Institute of Parasitic Diseases, Jiangsu Province and *An. stephensi* was provided by Professor Jingwen Wang’s laboratory at Fudan University. They were reared, colonized, and established as stable strains in the Laboratory of Pathogen Biology, Southern Medical University.

The rearing environment was maintained at a temperature of 26 °C ± 2 °C, humidity of 60% ± 10%, and a light cycle of 12 hours light:12 hours dark. Mosquito eggs were submerged in water 36–48 hours for hatching, and larval rearing density was strictly controlled. Larvae of *Culex* and *Aedes* were fed regularly with a quantified amount of turtle food (INCH-GOLD, Shenzhen, China) as required, while *Anopheles* larvae were fed with Tetra tropical fish food (Tetra Werke GmbH) until pupation. Adult mosquitoes were reared in cages and provided with 10% glucose solution. Female mosquitoes were fed with defibrinated sheep blood using a Hemotek membrane feeding system 3–7 days prior to oviposition.

### Wing-length measurements

Body size was represented by wing length, which serves as a reliable proxy for mosquito body size due to its low coefficient of variation. Wings were detached from the thorax and mounted on glass slides. Wing length was measured from the axillary incision to the apical margin (tip), excluding the wing fringe, using the Keyence VHX-7000 ultra-high-definition digital microscope (Keyence Co., Ltd., China). When mosquitoes are drawn into the Black Box, assuming similar flight speeds and body orientations, the duration of beam interruption at the infrared detection window can be used to identify insects. Therefore, wing length measurements provided critical data for validating the detection threshold of the Black Box.

Morphometric data for *An. gambiae* were provided by Professor Guiyun Yan’s research group at the University of California, Irvine. These measurements were performed following standardized protocols established in previous studies for *Ae. albopictus* and *Cx. quinquefasciatus*, where wing length was defined as the distance from the axillary incision to the apical margin, excluding the fringe [17].

### Recognition of *Anopheles* mosquitoes by the MS-300 detection window

This study aimed to evaluate the monitoring consistency of the MS-300 detection window for *Anopheles* mosquitoes by releasing test specimens into a controlled laboratory environment. Prior to experimentation, 3–7-day-old female *Ae. albopictus*, *An. sinensis*, and *An. anthropophagus* were starved for 6 h. Following cold anesthesia, 20 mosquitoes were introduced into the mosquito net. After 24 h, recordings were made of the number of mosquitoes captured in the MS-300 collection bag, dead mosquitoes on the ground, and the corresponding counts logged in the cloud database.

### Design of the Black Box

Based on previous research on the effectiveness of light, lures, photocatalysts, and other mosquito-attracting factors for *Aedes* and *Culex* mosquitoes, combined with the suitability of the MS-300 detection window [17], a device termed the “Black Box” was designed and fabricated using 3D printing.

### Laboratory tests

The laboratory environment was maintained at 27 ± 1 °C with a humidity of 60% ± 10%. A 1.5 m × 1.5 m × 1.5 m mosquito net was placed in the center of the room. The Black Box was placed in the center of the net, mounted on a custom-built iron stand 0.9 m high. Female mosquitoes aged 3–7 days were starved for 6 h prior to experiments, anesthetized by freezing, and then introduced into the mosquito net in batches of 20 individuals per experimental run. To standardize mosquito release procedures and avoid escape during handling, mosquitoes were briefly anesthetized via low-temperature treatment. Mosquitoes were collected once after 24 hours; no hourly collections were performed. After 24 hours, the number of mosquitoes captured in the Black Box trap bag and the number of dead mosquitoes on the ground were counted, and the number in the cloud database was recorded. The capture rate and consistency rate were calculated as below. Each species was tested over 5 separate days (n = 5 replicates).

- Capture rate (%) = number of mosquitoes recovered in the trap bag/ number of mosquitoes released × 100%
- Consistency rate (%) = number recorded by the cloud database/ number of mosquitoes recovered in the trap bag × 100%

### Semi-field tests

A semi-field screen house simulating the wild environment was used, with dimensions of 4 m × 3 m × 2.5 m, enclosed by iron screens, with natural temperature, light and good ventilation. The Black Box was placed in the center of the screen house and powered on. Prior to the experiment, 3–7-day-old female mosquitoes were starved for 6 hours. Each mosquito species was released separately; 100 individuals of a single species were released per 24-h experimental replicate. Mosquitoes were anesthetized by freezing before release. Mosquitoes were collected once after 24 hours; no hourly collections were performed. After 24 hours, the number of mosquitoes captured in the Black Box trap bag and the number of dead mosquitoes on the ground were counted, and the number in the cloud database was recorded. The trapping rate and consistency rate were calculated. The experiment was conducted from July to October 2023, with each species tested over 5 separate days (n = 5 replicates) at an ambient temperature of 25–31°C (average 28°C).

### Field tests

Field tests were conducted in two phases. The first phase aimed to compare the mosquito-trapping efficacy of the Black Box with traditional mosquito traps at different locations, evaluating the trapping effectiveness and automated counting capability of the Black Box for capturing *Anopheles* in field environments. The second phase aimed to determine the peak period of *Anopheles* capture (i.e., whether *Anopheles* are mainly captured during the day or night) and statistically analyze the blood-feeding status of field-captured *Anopheles*.

### Study sites

Phase 1: Two sites were established: Site A (a pig farm near Yanta Road) and Site B (a cattle farm near Honglin Road), both located in Meilan District, Haikou City, Hainan Province.
Phase 2: Conducted in Shanwei Village, Yanfeng Town, Meilan District, Haikou City, Hainan Province.
Phase 1 Protocol: At Sites A and B, Black Boxes were placed, and a Kongfu Xiaoshuai LED Mosquito Trap (Zhongshan Meitu Electric Appliance Co., Ltd.) was hung 10–20 m away at a height of 1.5 m. An experimental set was defined as one complete 24-h interval rather than as a single calendar date. Phase 1 comprised 31 consecutive 24-h sets. The recorded start dates ranged from the afternoon of August 4 to the afternoon of September 3, 2024; the final set ended on the afternoon of September 4, so the experiment spanned 32 calendar dates. The Black Box and LED trap were emptied once at the end of each complete set; no hourly manual collections were conducted. HLC was conducted for 30 min at approximately 7:00 AM and 7:00 PM at both sites. Because complete daily HLC records were unavailable for all sets and sampling effort differed from that of the continuously operating traps, HLC findings were summarized descriptively and excluded from formal inferential comparisons.
Phase 2 Protocol: An experimental set was defined by elapsed sampling time rather than by a single calendar date. Phase 2 comprised 36 consecutive 24-h sets. The start dates recorded in the dataset ranged from October 12 to November 16, 2024; because the final set ended at 7:00 PM on November 17, the experiment spanned 37 calendar dates. Each complete set comprised a daytime interval from 7:00 AM to 7:00 PM and a nighttime interval from 7:00 PM to 7:00 AM the following day, for a total of 72 twelve-hour observation intervals. Mosquitoes captured by the Black Box and LED trap were collected and enumerated at 7:00 AM and 7:00 PM, and the physical counts were verified against the cloud records.

### Statistical analysis

The trapping rates and consistency rates from the above experiments were statistically analyzed using SPSS 21.0 software (IBM, Chicago, USA). For the MS-300 and Black Box laboratory and semi-field experiments, consistency rates and capture rates from independent experimental replicates were compared among mosquito species using Kruskal-Wallis tests. When an overall test was significant, pairwise Mann-Whitney U tests with Holm adjustment were performed. Pooled rates were calculated from the corresponding total cloud and trap-bag counts and are reported descriptively, whereas inferential tests used the replicate-level rates. In Phase 1, Black Box and light-trap counts from the same 24-h experimental set were compared by genus using two-sided Wilcoxon signed-rank tests. HLC findings were summarized descriptively and excluded from inferential comparisons because complete effort-matched daily records were unavailable. In Phase 2, daytime and nighttime counts within the same 24-h experimental set were compared using a two-sided Wilcoxon signed-rank test. The first 16 and subsequent 20 sets were compared using two-sided Mann-Whitney U tests. All tests were two-sided, and *P* < 0.05 was considered statistically significant. The complete statistical results are summarized in Tables 2–4.

## Results

### Size measurement of malaria vector mosquitoes

Morphological characteristics vary among mosquito species. The average body length was measured in 3–7-day-old male and female adults of four malaria vectors (*An. sinensis*, *An. anthropophagus*, *An. stephensi*, and *An. gambiae*) (Table 1). The data showed that the average size of *Anopheles* mosquitoes was similar to that of *Ae. albopictus* (3.00 ± 0.017 mm) and *Cx. quinquefasciatus*(3.07 ± 0.016 mm), However, body size can vary with environmental and larval rearing conditions and should not be treated as a stand-alone feature for species identification. These measurements were used only to inform detection-window calibration, not to establish species identity.

**Table 1:** Average body length of 3–7-day-old of *Anopheles* mosquitoes.

Mosquito Species	Sex	Average Body Length (mm) ± SD	95% Confidence Interval
*An. sinensis*	Male	4.90 ± 0.33	4.80–5.01
	Female	5.17 ± 0.17	5.12–5.22
*An. anthropophagus*	Male	4.97 ± 0.23	4.92–5.07
	Female	5.09 ± 0.21	5.05–5.15
*An. stephensi*	Male	3.61 ± 0.26	3.55–3.71
	Female	3.81 ± 0.33	3.73–3.93
*An. gambiae*	Male	3.92 ± 0.24	3.84–3.99
	Female	4.08 ± 0.35	3.97–4.20

### Recognition of *Anopheles* mosquitoes by the MS-300 detection window

Previous studies have demonstrated that the MS-300 system enables real-time detection and monitoring of *Aedes* and *Culex* mosquitoes [17]. Building on this foundation, the present study compared the monitoring consistency of the MS-300 among the three test species. The result of mosquitoes in the MS-300 collection bag, dead mosquitoes on the ground, and the count recorded in the cloud database were documented (Fig 1A).

**Fig 1:**
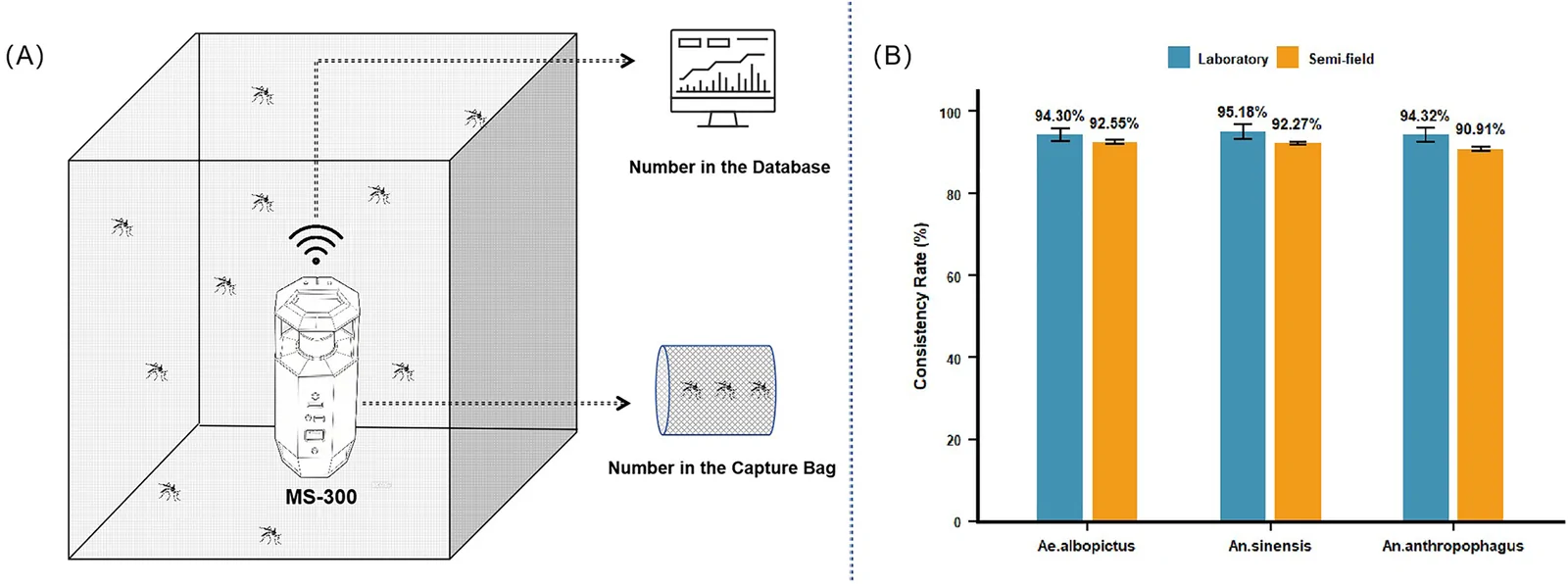
The consistency rates of the MS-300 in the laboratory and semi-field test. **(A)** MS-300 Monitoring Mode Diagram. **(B)** The results of the consistency rates of the MS-300 to *Ae. albopictus*, *An. sinensis* and *An. anthropophagus*.

Under laboratory conditions, the MS-300 exhibited high monitoring consistency. Consistency rates were 94.30% for *Ae. albopictus*, 95.18% for *An. sinensis*, and 94.32% for *An. anthropophagus*(Fig 1B). Consistency rates did not differ among the three species (Kruskal-Wallis *H* = 0.034, *df* = 2, *P* = 0.983; Table 2). All raw data are available in S1 Table.

**Table 2:** Statistical comparisons of MS-300 and Black Box performance in laboratory and semi-field experiments.

Outcome	Replicates	Pooled rate (%)a,b	Test statistic	*P* value	Post hoc result
MS-300 laboratory consistency	10 per species	94.30/ 95.18/ 94.32	*H* = 0.034; *df* = 2	0.983	Not significant
MS-300 semi-field consistency	6 per species	92.55/ 92.27/ 90.91	*H* = 1.747; *df* = 2	0.417	Not significant
Black Box Laboratory capture rate	5 per species	94.00/ 94.00/ 95.00	*H* = 0.234; *df* = 2	0.890	Not significant
Black Box Laboratory consistency rate	5 per species	98.94/ 98.94/ 97.89	*H* = 0.223; *df* = 2	0.894	Not significant
Black Box Semi-field capture rate	5 per species	73.60/ 90.80/ 83.00	*H* = 6.804; *df* = 2	0.033	*An. sinensis* vs *An. stephensi*: Holm-adjusted *P* = 0.024; other pairs not significant
Black Box Semi-field consistency rate	5 per species	92.12/ 94.27/ 93.73	*H* = 2.207; *df* = 2	0.332	Not significant

^a^For MS-300 rows, values are presented in the order *Ae. albopictus*/ *An. sinensis*/ *An. anthropophagus*.

^b^For Black Box rows, values are presented in the order *An. sinensis*/ *An. stephensi*/ *An. anthropophagus*.

We also conducted the tests to evaluate the monitoring consistency rate of the MS-300 for *Anopheles* mosquitoes in a semi-field environment. The results showed that under semi-field conditions, the monitoring consistency rates of the MS-300 for *Ae. albopictus*, *An. sinensis*, and *An. anthropophagus* were 92.55%, 92.27%, and 90.91%, respectively (Fig 1B). Consistency rates did not differ among species (Kruskal-Wallis *H* = 1.747, *df* = 2, *P* = 0.417; Table 2). The experimental data are presented in S2 Table.

### Design and 3D printing of the Black Box

Based on the resting habits of *Anopheles* mosquitoes, the test results of the MS-300 detection window, and the efficacy of multiple attractant elements on *Anopheles* [23–25], we designed and 3D printed a real-time monitoring device for malaria vectors,the Black Box (Fig 2A).

**Fig 2:**
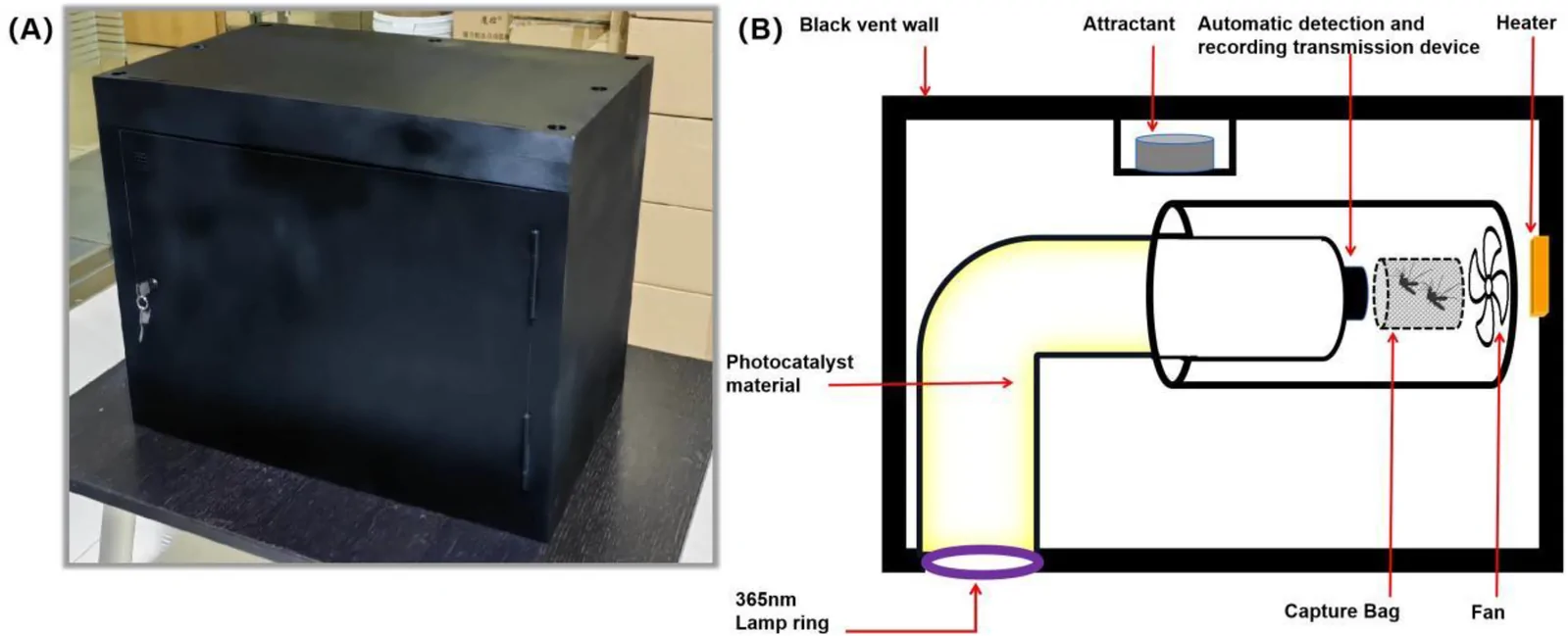
Schematic diagram of internal structure of the Black Box. **(A)** The Appearance of the Black Box. **(B)** The internal structure of the Black Box.

The device mainly consists of four components: a mosquito trapping module, a mosquito identification and sensing module, an automatic identification and wireless transmission module, and a mosquito storage module. Additionally, the Black Box is equipped with a thermometer, hygrometer, and photometer to collect real-time climate data at the mosquito capture site (Fig 2B).

The Black Box has dimensions of 55 cm in length, 40 cm in width, and 30 cm in height. The overall appearance is black, with the mosquito inlet designed to face downward. The black housing was retained from an earlier *Aedes*- and *Culex*-oriented design because host-seeking *Ae. albopictus* and *Cx. pipiens* showed strong responses to black or other dark visual targets under laboratory conditions [26]. Because this evidence did not directly evaluate the *Anopheles* species studied here, the independent contribution of housing color to *Anopheles* attraction was not assumed and was not tested in the present study. The device was adapted for *Anopheles* surveillance by combining the housing design with specific-wavelength light, thermal stimulation, a synthetic lure, and photocatalytic materials. The external design and internal structure of the Black Box are shown in Fig 2.

### Laboratory experiment on the monitoring efficacy of the Black Box for *Anopheles* mosquitoes

The assembled Black Box was placed in a laboratory environment (Fig 3A left). Twenty test mosquitoes were released daily to test the efficacy of the device on three malaria vectors: *An. sinensis*, *An. stephensi*, and *An. anthropophagus*.

**Fig 3:**
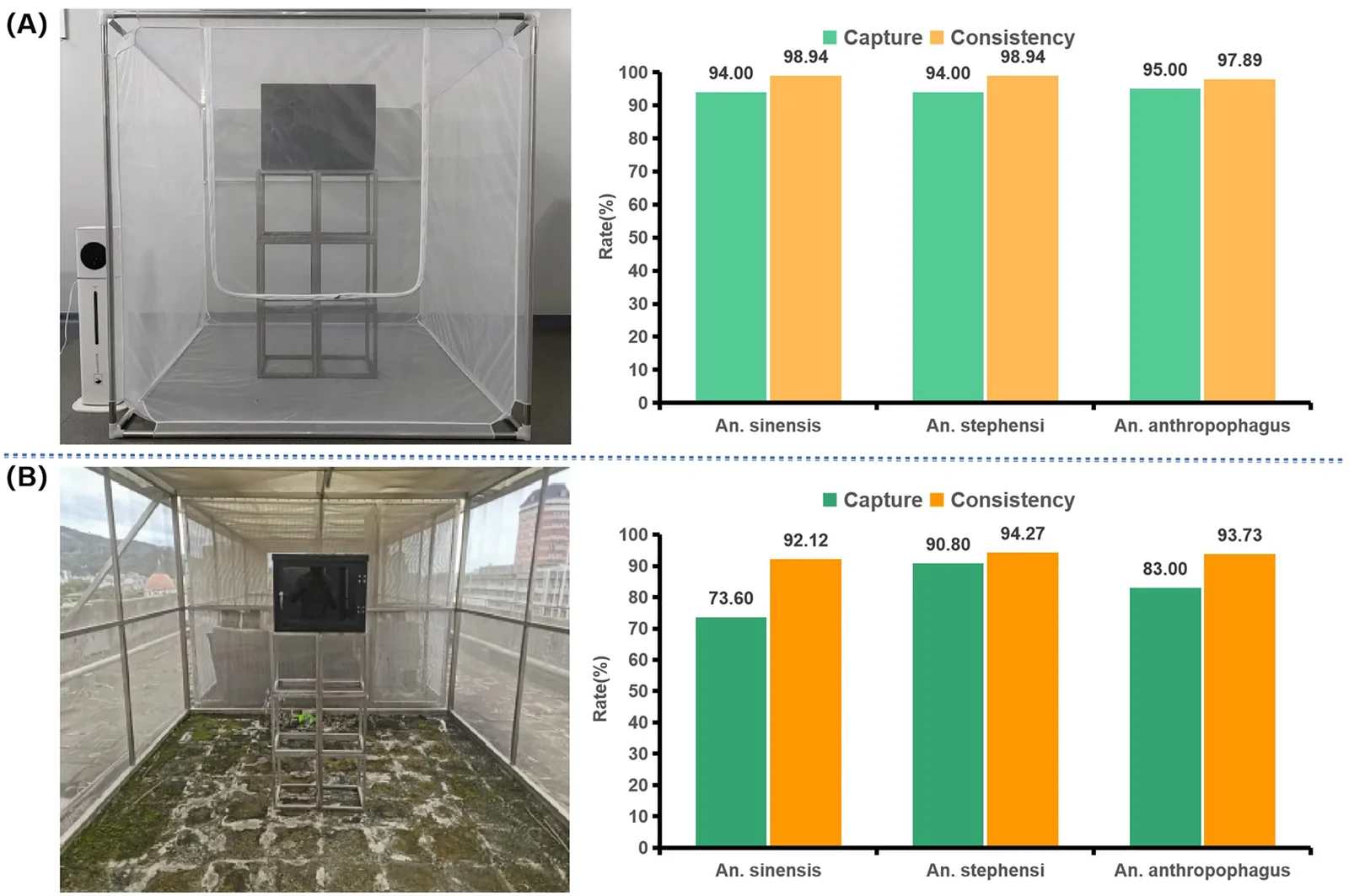
Testing of the Black Box in laboratory and semi-field. **(A)** The experimental site in laboratory and the capture rate and consistency rate results of the Black Box on *Anopheles* mosquitoes in laboratory test. **(B)** The experimental site in semi-field and the capture rate and consistency rate results of the Black Box on *Anopheles* mosquitoes in semi-field test.

The average capture rates of the Black Box were 94.00% for *An. sinensis*, 94.00% for *An. stephensi*, and 95.00% for *An. anthropophagus*. Capture rates did not differ significantly among the three species (Kruskal-Wallis *H* = 0.234, *df* = 2, *P* = 0.890; Table 2). The average consistency rates between the number of mosquitoes captured in the trap bag and the data recorded in the cloud database were 98.94%, 98.94%, and 97.89% respectively (Fig 3A, right). All capture and consistency rates exceeded 90%. Consistency rates did not differ significantly among species (Kruskal-Wallis *H* = 0.223, *df* = 2, *P* = 0.894). The experimental data are presented in S3 Table.

### Semi-field experiment on the monitoring efficacy of the Black Box for *Anopheles* mosquitoes

The assembled Black Box was placed in a semi-field experimental environment, with 100 *Anopheles* mosquitoes released daily to test its efficacy on three malaria vector species: *An. sinensis*, *An. stephensi*, and *An. anthropophagus*. The experimental site is shown in Fig 3B, left. The average capture rates of the Black Box for *An. sinensis*, *An. stephensi*, and *An. anthropophagus* in the semi-field setting were 73.60%, 90.80%, and 83.00%, respectively. Capture rates differed among species (Kruskal-Wallis *H* = 6.804, *df* = 2, *P* = 0.033); the Holm-adjusted comparison showed a lower rate for *An. sinensis* than for *An. stephensi* (adjusted *P* = 0.024), whereas the other pairwise comparisons were not significant. The average consistency rates between the number captured in the trap bag and the cloud records were 92.12%, 94.27%, and 93.73%, respectively, with no significant difference among species (Kruskal-Wallis *H* = 2.207, *df* = 2, *P* = 0.332; Table 2) (Fig 3B, right). The relatively lower trapping rates for *An. sinensis* and *An. anthropophagus* may be attributed to the fact that laboratory-reared *Anopheles* mosquitoes were not adapted to the semi-field climatic conditions, resulting in reduced vitality and a higher number of deaths, which in turn led to lower trapping rates of the Black Box. The experimental data are presented in S3 Table.

### Field test of Black Box on the trapping efficacy for *Anopheles* mosquitoes

#### Phase-1 experiment.

During 31 consecutive 24-h field experimental sets (Fig 4A–4F), the Black Box captured a total of 5,109 mosquitoes (excluding insects in the “other” category), mainly including species such as *Ae. albopictus*, *Cx. quinquefasciatus*, and *An. sinensis*. Given that this device is designed specifically for trapping malaria-transmitting *Anopheles* vectors, subsequent analyses primarily focused on the capture performance for *Anopheles* mosquitoes, despite the incidental capture of *Aedes* and *Culex* species. As shown in S4 Table, the consistency rate between the actual mosquito counts trapped by the Black Box and the automated counts recorded in the cloud database reached 94.17%.

**Fig 4:**
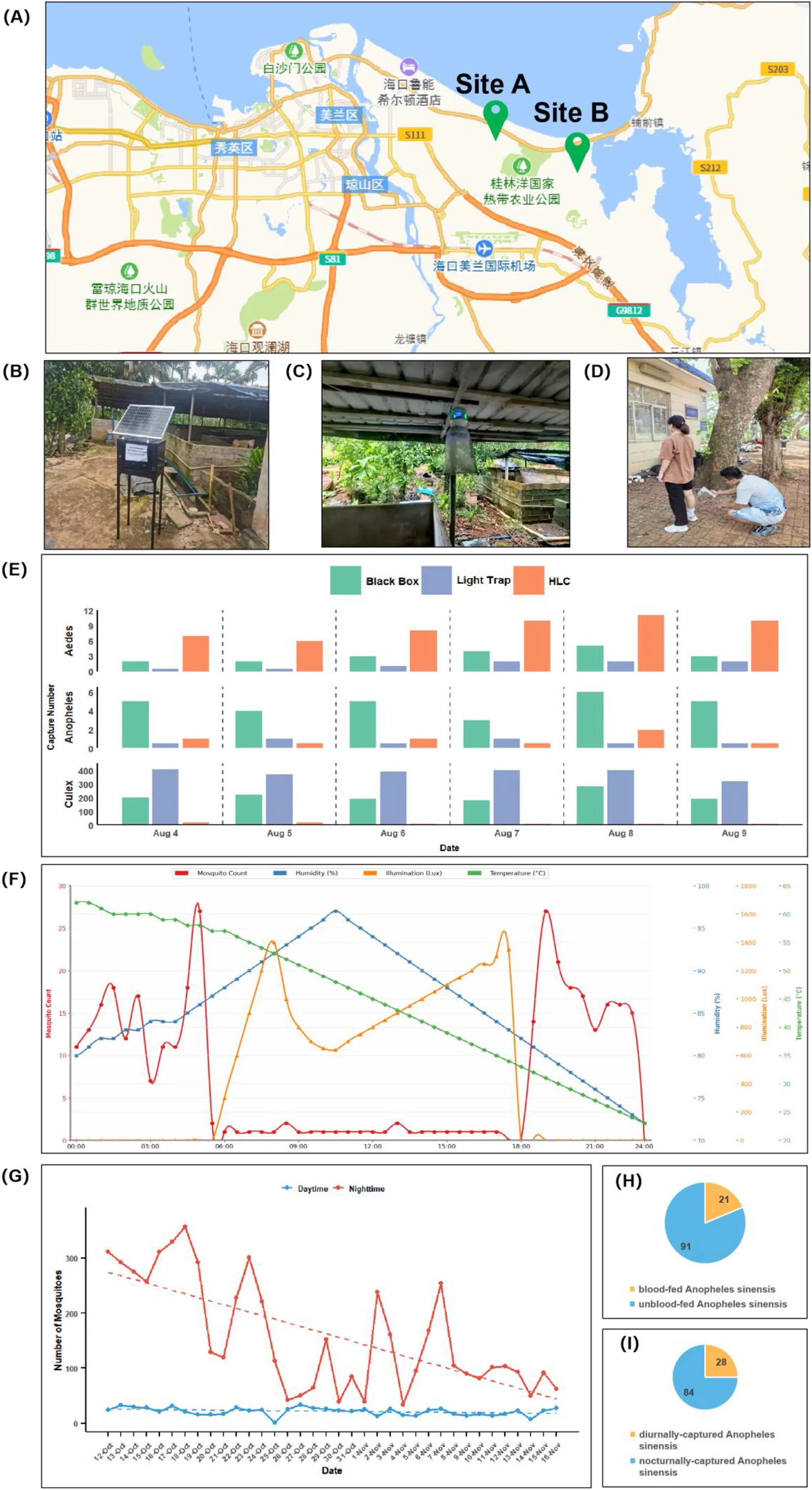
Testing of the Black Box in the field. **(A)** satellite image of test point. **(B)** Schematic diagram of the Black Box monitoring. **(C)** Schematic diagram of Light trap. **(D)** Schematic diagram of HLC. **(E)** The captured *Aedes*, *Culex* and *Anopheles* mosquitoes by HLC, Light trap and Black Box from Aug. 4 to 9, 2024. **(F)** The mosquito numbers and relevant climate factors documented per half hour in the database. **(G)** Captured mosquitoes in daytime group and nighttime group from Oct. 12 to Nov. 17, 2024 (start dates through Nov. 16). **(H)** The blood-fed and unblood-fed numbers of captured *An. sinensis*. **(I)** Proportion of *An. sinensis* captured in diurnal and nocturnal periods.

Across the 31 paired 24-h experimental sets, the Black Box captured more *Aedes* mosquitoes than the light trap (mean ± SD, 1.42 ± 0.50 vs 0.13 ± 0.50; Wilcoxon *W* = 19.0, *P* < 0.001), fewer *Culex* mosquitoes (156.23 ± 30.70 vs 229.13 ± 89.76; *W* = 94.0, *P* = 0.002), and more *Anopheles* mosquitoes (7.16 ± 1.71 vs 0.90 ± 0.30; *W* = 0, *P* < 0.001; Table 3). HLC observations were summarized descriptively and were not included in inferential comparisons because complete paired daily HLC records were unavailable and sampling effort differed among methods (Fig 4E).

**Table 3:** Paired comparisons of Black Box and light-trap mosquito counts in Phase 1.

Mosquito genus	Sample size	Black Box vs light trap, mean ± SD	Wilcoxon statistic	*P* value	Result
*Aedes*	31 paired sets	1.42 ± 0.50 Vs 0.13 ± 0.50	*W* = 19.0	<0.001	Black Box higher
*Culex*	31 paired sets	156.23 ± 30.70 Vs 229.13 ± 89.76	*W* = 94.0	0.002	Light trap higher
*Anopheles*	31 paired sets	7.16 ± 1.71 Vs 0.90 ± 0.30	*W* = 0	<0.001	Black Box higher

Real-time trapping data from the database showed two peaks in the daily activity of mosquitoes, corresponding to sunrise and sunset respectively, which is consistent with the activity characteristics of mosquitoes (Fig 4F).

#### Phase-2 experiment.

To determine the peak period for *Anopheles* capture, i.e., to identify whether *Anopheles* mosquitoes are mainly captured during the day or at night, the number of mosquitoes captured in the bag of the Black Box and documented in the cloud database were counted every 12 hours for 36 consecutive sets with start dates from October 12 to November 16, 2024; the final nighttime interval ended on November 17. The experimental data are presented in S5 Table. Counts from 7:00 AM to 7:00 PM were recorded as the daytime group, and those from 7:00 PM to 7:00 AM were recorded as the nighttime group.

Across 36 complete paired sampling sets collected over 37 calendar dates, the mean number of mosquitoes captured during daytime periods was 21.28 ± 7.23, compared with 159.44 ± 101.73 during nighttime periods. The nighttime count was significantly higher (Wilcoxon signed-rank test, *W* = 0, *P* < 0.001; Table 4). During the first 16 sampling sets, mean daytime and nighttime counts were 23.19 ± 8.17 and 227.19 ± 102.87, respectively. In the subsequent 20 sampling sets, both daytime and nighttime capture numbers decreased: the average nighttime capture number was 105.25 ± 61.26, a decrease of 53.67% compared with the first 16 sampling sets (Mann-Whitney *U* = 268.0, *P* < 0.001); the average daytime capture number was 19.75 ± 6.15, a decrease of 14.82%, which was not statistically significant (*U* = 210.5, *P* = 0.111; Table 4) (Fig 4G).

**Table 4:** Statistical comparisons of Phase 2 daytime and nighttime mosquito counts.

Comparison	Sample size	Mean ± SD	Test statistic	*P* value	Result
Night vs day	36 paired sets	159.44 ± 101.73 vs 21.28 ± 7.23	Wilcoxon signed-rank; *W* = 0	<0.001	Night counts higher
Night: first 16 vs subsequent 20	16 vs 20	227.19 ± 102.87 vs 105.25 ± 61.26	Mann-Whitney; *U* = 268.0	<0.001	53.67% reduction
Day: first 16 vs subsequent 20	16 vs 20	23.19 ± 8.17 vs 19.75 ± 6.15	Mann-Whitney; *U* = 210.5	0.111	14.82% reduction; not significant

Across the 36 Phase 2 experimental sets spanning 37 calendar dates, a total of 112 female *An. sinensis* were captured, including 28 during daytime and 84 at nighttime (Fig 4I). Furthermore, 21 blood-fed individuals were collected outdoors, accounting for 18.75% of the total outdoor captures (Fig 4H).

## Discussion

Based on the resting habits of *Anopheles* mosquitoes, combined with the MS-300 detection window’s capability to identify common malaria vectors and the established attractant effects of light, heat, odor, and CO_2_ [25], we developed the Black Box, an internet-based device for the efficient trapping and real-time monitoring of malaria vectors.

In laboratory assays, the mean trapping rates of the Black Box for *An. sinensis*, *An. stephensi*, and *An. anthropophagus*, as well as the consistency rates with cloud-based data, all exceeded 90.00%. This confirms the device’s fundamental functionality under controlled conditions. However, under semi-field conditions, trapping rates decreased markedly, particularly for *An. anthropophagus* and *An. sinensis*. Unlike laboratory settings, the open semi-field environment exposes mosquitoes to natural convective airflow. *Anopheles* species rely heavily on precise thermal plumes and CO_2_ gradients to locate hosts; these cues are readily dispersed by ambient wind, diminishing the effective attraction range of the traps [27–33]. Furthermore, the absence of supplemental nutrition (sucrose solution) in the semi-field setup, necessitated by the experimental design to standardize hunger levels, likely contributed to reduced vigor or mortality. These factors highlight a limitation in transitioning from still-air indoor labs to dynamic outdoor environments, rather than a specific failure of the Black Box hardware. Despite the reduced capture numbers, the consistency rates between trapped counts and database records remained above 90%, validating the device’s real-time data transmission and quantification accuracy.

In Phase 1 field experiments, the consistency rate between cloud database records and physical captures reached 94.17%, demonstrating robust monitoring performance across common vectors. Comparative analysis revealed distinct efficacy profiles among the Black Box, light traps, and HLC. Light traps captured more *Cx. quinquefasciatus* than the Black Box or HLC in the available field observations; this comparison was descriptive because complete effort-matched HLC records were unavailable. This is likely attributable to the strong phototactic response of *Culex* to the intense illumination of light traps [34–36], whereas the Black Box utilizes low-intensity light strips at the inlet. Conversely, HLC primarily captured *Ae. albopictus*. Given that *Ae. albopictus* exhibits bimodal crepuscular activity [37,38], while *Culex* and *Anopheles* are predominantly nocturnal [36,39–43], the timing of HLC collections (7:00 AM to 7:00 PM) inherently missed the peak activity windows of *Culex* and *Anopheles*, explaining the lower capture rates for these genera via HLC.

The Black Box demonstrated competent trapping efficacy for *Aedes*, *Culex*, and *Anopheles*. Descriptive field observations indicated higher *Anopheles* counts in the Black Box than in the illustrated light-trap and HLC observations; however, no inferential comparison involving HLC was made because complete effort-matched daily data were unavailable. This superiority is likely due to the integration of multisensory lures: beyond ultraviolet light attraction, the device incorporates a specific synthetic attractant for malaria vectors, a thermal module simulating human skin temperature, and a photocatalyst (e.g., titanium dioxide, TiO_2_) that generates CO_2_ through the photo-degradation of organic matter. This multidimensional approach enhances host mimicry, thereby increasing trapping efficiency over single-modality traps.

Diel activity analysis revealed bimodal peaks corresponding to sunrise and sunset, consistent with known mosquito circadian rhythms. The morning peak was significantly lower than the evening peak. While the evening peak comprises *Aedes, Culex*, and *Anopheles*, the lower morning peak is likely driven by physiological and environmental factors rather than mere species composition. Ambient temperatures during pre-sunrise hours are typically lower, reducing mosquito metabolic rates and flight activity [44,45]. Additionally, females that completed blood meals during the night may exhibit post-prandial quiescence in the early morning, further contributing to reduced trap captures during this period.

Across the 36 Phase 2 experimental sets spanning 37 calendar dates, capture trends correlated strongly with meteorological conditions. Periods of low capture coincided precisely with periods of precipitation, as confirmed by local weather data [46–48]. Rainfall physically impedes mosquito flight and increases mortality risk, leading to reduced activity [49]. Following each rainy period, a marked rebound in capture rates was observed. This phenomenon aligns with established entomological principles: rising ambient humidity reduces desiccation stress, newly formed breeding pools stimulate oviposition site seeking, and females temporarily suppressed by rain engage in compensatory host-seeking flights [50].

In the latter 20 days of Phase 2, capture numbers declined significantly compared to the first 16 days. This trend likely reflects seasonal population dynamics, specifically a decrease in ambient temperatures [51], coupled with a localized depletion of the mosquito population due to continuous trapping at a fixed site.

Throughout Phase 2, 112 female *An. sinensis* were captured (mean: 3.11 per day). This figure was lower than that of *Cx. quinquefasciatus* and *Ae. albopictus*. In Hainan, China, *An. sinensis* typically peaks between July and September [52]. Since Phase 2 was conducted from October to November, the observed decline is consistent with the expected seasonal recession of this species.

Analysis of the 112 *An. sinensis* specimens showed that 21 (18.75%; exact 95% CI, 12.60%-26.97%) were blood-fed. The capture of engorged mosquitoes in outdoor traps suggests either outdoor biting activity or exophilic resting following indoor feeding. While *An. sinensis* is traditionally characterized by endophagic feeding and exophilic resting, our observations suggest potential behavioral plasticity in this population [53,54]. Regarding diel distribution, 75.0% of *An. sinensis* were captured at night and 25.0% during the day (7:00 AM–7:00 PM). Although this confirms predominant nocturnal activity, the inclusion of the crepuscular period (sunset) within the daytime sampling window complicates the interpretation of diurnal activity. Further studies are required to confirm unequivocal daytime biting behavior.

Despite the promising performance of the Black Box, several limitations should be acknowledged. First, the device cannot automatically distinguish among *Anopheles* species, requiring manual morphological identification. Future studies should explore whether additional optical, image-based, or machine-learning approaches can support species-level identification; these potential upgrades remain prospective and were not developed or validated in the present study. Second, field validation was restricted to a single tropical region (Hainan Province) in October–November, missing the peak season and lacking broader geographic representativeness. Third, semi-field trapping rates were lower than laboratory results, likely due to the maladaptation of laboratory-reared mosquitoes to outdoor microclimates; wild populations remain untested. Finally, while outdoor blood-feeding and daytime activity were observed, the underlying behavioral mechanisms and the impacts of extreme weather or vegetation require systematic assessment.

## Conclusions

This study innovatively developed an internet-based efficient trapping and real-time monitoring device for malaria vectors, the Black Box. Laboratory, semi-field, and field tests demonstrated that it can efficiently trap and monitor common malaria vectors, including *An. sinensis*, *An. anthropophagus* and, *An. stephensi*, showing promise for application in malaria vector surveillance. A limitation of the device is its inability to distinguish between different species of vector mosquitoes, which requires further research and improvement.

## Supporting information

S1 Table Detection Effect of MS-300 Monitor on *Ae. albopictus*, *An. sinensis*, and *An. anthropophagus* in laboratory. (XLSX)

S2 Table Detection Effect of MS-300 Monitor on *Ae. albopictus*, *An. sinensi*s, and *An. anthropophagus* in semi-field. (XLSX)

S3 Table The result of Black Box on the monitoring efficacy for *Anopheles* mosquitoes. (XLSX)

S4 Table The results of Black Box in Phase I Field Trial (from August 4 to September 3, 2024). (XLSX)

S5 Table Daytime and nighttime mosquito counts during the Phase 2 field experiment. (XLSX)

## References

[1] Zhao T, Xue R-D. Vector biology and integrated management of malaria vectors in China. Annu Rev Entomol. 2024;69:333–54. doi: 10.1146/annurev-ento-021323-085255 38270986

[2] Jiang X, Peery A, Hall AB, Sharma A, Chen X-G, Waterhouse RM, et al. Genome analysis of a major urban malaria vector mosquito, *Anopheles stephensi*. Genome Biol. 2014;15(9):459. doi: 10.1186/s13059-014-0459-2 25244985 PMC4195908

[3] Takken W, Charlwood D, Lindsay SW. The behaviour of adult *Anopheles gambiae*, sub-Saharan Africa’s principal malaria vector, and its relevance to malaria control: A review. Malar J. 2024;23(1):161. doi: 10.1186/s12936-024-04982-3 38783348 PMC11112813

[4] Kogan F. Remote sensing for malaria: Monitoring and predicting malaria from operational satellites. Remote Sens Malar. 2020.

[5] Nalinya S, Musoke D, Deane K. Malaria prevention interventions beyond long-lasting insecticidal nets and indoor residual spraying in low- and middle-income countries: A scoping review. Malar J. 2022;21(1):31. doi: 10.1186/s12936-022-04052-6 35109848 PMC8812253

[6] Okumu FO, Mbeyela E, Lingamba G, Moore J, Ntamatungiro AJ, Kavishe DR, et al. Comparative field evaluation of combinations of long-lasting insecticide treated nets and indoor residual spraying, relative to either method alone, for malaria prevention in an area where the main vector is *Anopheles arabiensis*. Parasit Vectors. 2013;6:46. doi: 10.1186/1756-3305-6-46 23433393 PMC3606331

[7] Killeen GF, Kihonda J, Lyimo E, Oketch FR, Kotas ME, Mathenge E, et al. Quantifying behavioural interactions between humans and mosquitoes: Evaluating the protective efficacy of insecticidal nets against malaria transmission in rural Tanzania. BMC Infect Dis. 2006;6:161. doi: 10.1186/1471-2334-6-161 17096840 PMC1657018

[8] Mwangangi JM, Mbogo CM, Orindi BO, Muturi EJ, Midega JT, Nzovu J, et al. Shifts in malaria vector species composition and transmission dynamics along the Kenyan coast over the past 20 years. Malar J. 2013;12:13. doi: 10.1186/1475-2875-12-13 23297732 PMC3544599

[9] Bayoh MN, Mathias DK, Odiere MR, Mutuku FM, Kamau L, Gimnig JE, et al. *Anopheles gambiae*: Historical population decline associated with regional distribution of insecticide-treated bed nets in western Nyanza Province, Kenya. Malar J. 2010;9:62. doi: 10.1186/1475-2875-9-62 20187956 PMC2838909

[10] Govella NJ, Chaki PP, Mpangile JM, Killeen GF. Monitoring mosquitoes in urban Dar es Salaam: Evaluation of resting boxes, window exit traps, CDC light traps, Ifakara tent traps and human landing catches. Parasit Vectors. 2011;4:40. doi: 10.1186/1756-3305-4-40 21418622 PMC3069960

[11] Mawejje HD, Asiimwe JR, Kyagamba P, Kamya MR, Rosenthal PJ, Lines J, et al. Impact of different mosquito collection methods on indicators of *Anopheles* malaria vectors in Uganda. Malar J. 2022;21(1):388. doi: 10.1186/s12936-022-04413-1 36536428 PMC9761930

[12] Farlow R, Russell TL, Burkot TR. Nextgen Vector Surveillance Tools: Sensitive, specific, cost-effective and epidemiologically relevant. Malar J. 2020;19(1):432. doi: 10.1186/s12936-020-03494-0 33239015 PMC7687713

[13] Krajacich BJ, Slade JR, Mulligan RT, Labrecque B, Kobylinski KC, Gray M, et al. Design and testing of a novel, protective human-baited tent trap for the collection of anthropophilic disease vectors. J Med Entomol. 2014;51(1):253–63. doi: 10.1603/me13090 24605476 PMC4106152

[14] González-Pérez MI, Faulhaber B, Aranda C, Williams M, Villalonga P, Silva M, et al. Field evaluation of an automated mosquito surveillance system which classifies *Aedes* and *Culex* mosquitoes by genus and sex. Parasit Vectors. 2024;17(1):97. doi: 10.1186/s13071-024-06177-w 38424626 PMC10905882

[15] Silva M, Gouveia BR, Santos JM, Guerreiro N, Monteiro A, Almeida S, et al. Enhancing entomological surveillance: Real-time monitoring of mosquito activity with the VECTRACK system in rural and urban areas. Biology (Basel). 2025;14(8):1047. doi: 10.3390/biology14081047 40906386 PMC12383513

[16] Eckert J, Oladipupo S, Wang Y, Jiang S, Patil V, McKenzie BA, et al. Which trap is best? Alternatives to outdoor human landing catches for malaria vector surveillance: A meta-analysis. Malar J. 2022;21(1):378. doi: 10.1186/s12936-022-04332-1 36494724 PMC9733232

[17] Lai Z, Wu J, Xiao X, Xie L, Liu T, Zhou J, et al. Development and evaluation of an efficient and real-time monitoring system for the vector mosquitoes, *Aedes albopictus* and *Culex quinquefasciatus*. PLoS Negl Trop Dis. 2022;16(9):e0010701. doi: 10.1371/journal.pntd.0010701 36074773 PMC9455839

[18] Palaniyandi M, Anand PH, Maniyosai R, Mariappan T, Das PK. The integrated remote sensing and GIS for mapping of potential vector breeding habitats, and the internet GIS surveillance for epidemic transmission control, and management. J Entomol Zool Stud. 2016;4(2):310–8.

[19] Palaniyandi M. Web mapping GIS: GPS under the GIS umbrella for Aedes species dengue and chikungunya vector mosquito surveillance and control. Int J Mosq Res. 2014;1(3):18–25.

[20] Oliveira D, Mafra S. Implementation of an intelligent trap for effective monitoring and control of the *Aedes aegypti* Mosquito. Sensors (Basel). 2024;24(21):6932. doi: 10.3390/s24216932 39517830 PMC11548326

[21] Vasconcelos D, Yin MS, Wetjen F, Herbst A, Ziemer T, Frster A, et al. Counting mosquitoes in the wild: An internet of things approach. Proc Conf Inf Technol Soc Good; 2021.

[22] Javed N, Paradkar PN, Bhatti A. An overview of technologies available to monitor behaviours of mosquitoes. Acta Trop. 2024;258:107347. doi: 10.1016/j.actatropica.2024.107347 39103110

[23] Konopka JK, Task D, Afify A, Raji J, Deibel K, Maguire S, et al. Olfaction in *Anopheles* mosquitoes. Chem Senses. 2021;46:bjab021. doi: 10.1093/chemse/bjab021 33885760 PMC8256107

[24] Day CA, Richards SL, Reiskind MH, Doyle MS, Byrd BD. Context-dependent accuracy of the BG-Counter remote mosquito surveillance device in North Carolina. J Am Mosq Control Assoc. 2020;36(2):74–80. doi: 10.2987/19-6903.1 33647123

[25] Wu J, Cai L, Hu K, Cai Y, Lai Z, Zhao S, et al. Design and evaluation of an efficient mosquito trap. Pest Manag Sci. 2024;80(10):5200–11. doi: 10.1002/ps.8247 38873906

[26] Jung SH, Kim D, Jung K-S, Lee D-K. Color preference for host-seeking activity of Aedes albopictus and Culex pipiens (Diptera: Culicidae). J Med Entomol. 2021;58(6):2446–52. doi: 10.1093/jme/tjab100 34104944

[27] Hoffmann EJ, Miller JR. Reassessment of the role and utility of wind in suppression of mosquito (Diptera: Culicidae) host finding: Stimulus dilution supported over flight limitation. J Med Entomol. 2003;40(5):607–14. doi: 10.1603/0022-2585-40.5.607 14596273

[28] Bidlingmayer WL, Day JF, Evans DG. Effect of wind velocity on suction trap catches of some Florida mosquitoes. J Am Mosq Control Assoc. 1995;11(3):295–301. 8551296

[29] Cummins B, Cortez R, Foppa IM, Walbeck J, Hyman JM. A spatial model of mosquito host-seeking behavior. PLoS Comput Biol. 2012;8(5):e1002500. doi: 10.1371/journal.pcbi.1002500 22615546 PMC3355069

[30] Spitzen J, Spoor CW, Grieco F, ter Braak C, Beeuwkes J, van Brugge SP, et al. A 3D analysis of flight behavior of *Anopheles gambiae* sensu stricto malaria mosquitoes in response to human odor and heat. PLoS One. 2013;8(5):e62995. doi: 10.1371/journal.pone.0062995 23658792 PMC3642193

[31] Sutcliffe JF, Yin S. Effects of indoor air movement and ambient temperature on mosquito (*Anopheles gambiae*) behaviour around bed nets: Implications for malaria prevention initiatives. Malar J. 2021;20(1):427. doi: 10.1186/s12936-021-03957-y 34717652 PMC8557611

[32] Cribellier A, Spitzen J, Fairbairn H, van de Geer C, van Leeuwen JL, Muijres FT. Lure, retain, and catch malaria mosquitoes. How heat and humidity improve odour-baited trap performance. Malar J. 2020;19(1):357. 10.1186/s12936-020-03403-5 PMC7542916 33028362

[33] Liu Q, Liu X, Zhou G, Jiang J, Guo Y, Ren D, et al. Dispersal range of *Anopheles sinensis* in Yongcheng City, China by mark-release-recapture methods. PLoS One. 2012;7(11):e51209. doi: 10.1371/journal.pone.0051209 23226489 PMC3511368

[34] Pimnon S, Ngoen-Klan R, Sumarnrote A, Chareonviriyaphap T. UV light-emitting-diode traps for collecting nocturnal biting mosquitoes in urban Bangkok. Insects. 2022;13(6):526. doi: 10.3390/insects13060526 35735863 PMC9225645

[35] Suom C, Ginsberg HS, Bernick A, Klein C, Buckley PA, Salvatore C, et al. Host-seeking activity and avian host preferences of mosquitoes associated with West Nile virus transmission in the northeastern U.S.A. J Vector Ecol. 2010;35(1):69–74. 10.1111/j.1948-7134.2010.00030.x 20618650

[36] Liu S, Zhou J, Kong L, Cai Y, Liu H, Xie Z, et al. Clock genes regulate mating activity rhythms in the vector mosquitoes, *Aedes albopictus* and *Culex quinquefasciatus*. PLoS Negl Trop Dis. 2022;16(12):e0010965. doi: 10.1371/journal.pntd.0010965 36455055 PMC9746994

[37] Lima-Camara TN, Lima JBP, Bruno RV, Peixoto AA. Effects of insemination and blood-feeding on locomotor activity of *Aedes albopictus* and *Aedes aegypti* (Diptera: *Culicidae*) females under laboratory conditions. Parasit Vectors. 2014;7:304. doi: 10.1186/1756-3305-7-304 24990394 PMC4094679

[38] Unlu I, Faraji A, Indelicato N, McNelly JR. Do tigers hunt during the day? Diel activity of the Asian tiger mosquito, *Aedes albopictus* (Diptera: *Culicidae*), in urban and suburban habitats of North America. PLoS Negl Trop Dis. 2021;15(8):e0009438. doi: 10.1371/journal.pntd.0009438 34415918 PMC8409682

[39] Dambach P, Schleicher M, Korir P, Ouedraogo S, Dambach J, Sié A, et al. Nightly biting cycles of *Anopheles* species in rural Northwestern Burkina Faso. J Med Entomol. 2018;55(4):1027–34. doi: 10.1093/jme/tjy043 29635478 PMC6025195

[40] Wang G, Vega-Rodríguez J, Diabate A, Liu J, Cui C, Nignan C, et al. Clock genes and environmental cues coordinate *Anopheles* pheromone synthesis, swarming, and mating. Science. 2021;371(6527):411–5. doi: 10.1126/science.abd4359 33479155 PMC9854397

[41] Wilke ABB, Mhlanga A, Kummer AG, Vasquez C, Moreno M, Petrie WD, et al. Diel activity patterns of vector mosquito species in the urban environment: Implications for vector control strategies. PLoS Negl Trop Dis. 2023;17(1):e0011074. doi: 10.1371/journal.pntd.0011074 36701264 PMC9879453

[42] Khatib B, Mcha J, Pandu Z, Haji M, Hassan M, Ali H, et al. Early evening outdoor biting by malaria-infected *Anopheles arabiensis* vectors threatens malaria elimination efforts in Zanzibar. Malar J. 2025;24(1):92. doi: 10.1186/s12936-025-05333-6 40114159 PMC11927253

[43] Rowland M. Changes in the circadian flight activity of the mosquito *Anopheles stephensi* associated with insemination, blood‐feeding, oviposition and nocturnal light intensity. Physiol Entomol. 1989;14(1):77–84. doi: 10.1111/j.1365-3032.1989.tb00939.x

[44] Rund SSC, O’Donnell AJ, Prior KF, van der Veen DR. Seasonal plasticity in daily timing of flight activity in *Anopheles stephensi* is driven by temperature modulation of dawn entrainment. Philos Trans R Soc Lond B Biol Sci. 2025;380(1918):20230343. 10.1098/rstb.2023.0343 PMC11753879 39842479

[45] Chow WK, Beebe NW, Ambrose L, Pickering P, Cooper RD. Seasonal assessment on the effects of time of night, temperature and humidity on the biting profile of *Anopheles farauti* in north Queensland, Australia using a population naive to malaria vector control pressures. Malar J. 2023;22(1):85. 10.1186/s12936-023-04495-5 PMC9996873 36890552

[46] Charlwood JD, Braganca M. The effect of rainstorms on adult *Anopheles funestus* behavior and survival. J Vector Ecol. 2012;37(1):252–6. doi: 10.1111/j.1948-7134.2012.00224.x 22548561

[47] Degaetano AT. Meteorological effects on adult mosquito (*Culex*) populations in metropolitan New Jersey. Int J Biometeorol. 2005;49(5):345–53. doi: 10.1007/s00484-004-0242-2 15864404

[48] Meyrowitsch DW, Pedersen EM, Alifrangis M, Scheike TH, Malecela MN, Magesa SM, et al. Is the current decline in malaria burden in sub-Saharan Africa due to a decrease in vector population? Malar J. 2011;10:188. doi: 10.1186/1475-2875-10-188 21752273 PMC3160426

[49] Dickerson AK, Shankles PG, Madhavan NM, Hu DL. Mosquitoes survive raindrop collisions by virtue of their low mass. Proc Natl Acad Sci U S A. 2012;109(25):9822–7. doi: 10.1073/pnas.1205446109 22665779 PMC3382494

[50] Karki S, Hamer GL, Anderson TK, Goldberg TL, Kitron UD, Krebs BL, et al. Effect of trapping methods, weather, and landscape on estimates of the *Culex* vector mosquito abundance. Environ Health Insights. 2016;10:93–103. doi: 10.4137/EHI.S33384 27375359 PMC4918690

[51] Reinhold JM, Lazzari CR, Lahondère C. Effects of the environmental temperature on *Aedes aegypti* and *Aedes albopictus* mosquitoes: A review. Insects. 2018;9(4):158. 10.3390/insects9040158 PMC6316560 30404142

[52] Feng X, Zhang S, Huang F, Zhang L, Feng J, Xia Z, et al. Biology, bionomics and molecular biology of Anopheles sinensis Wiedemann 1828 (Diptera: *Culicidae*), main malaria vector in China. Front Microbiol. 2017;8:1473. 10.3389/fmicb.2017.01473 PMC5552724 28848504

[53] Zhang S, Guo S, Feng X, Afelt A, Frutos R, Zhou S, et al. *Anopheles* vectors in Mainland China while approaching malaria elimination. Trends Parasitol. 2017;33(11):889–900. doi: 10.1016/j.pt.2017.06.010 28734898

[54] Li Z, Roux E, Dessay N, Girod R, Stefani A, Nacher M, et al. Mapping a knowledge-based malaria hazard index related to landscape using remote sensing: Application to the cross-border area between French Guiana and Brazil. Remote Sens. 2016;8(4):319. doi: 10.3390/rs8040319

